# Modulation of the long non-coding RNA *Mir155hg* by high, but not moderate, hydrostatic pressure in cartilage precursor cells

**DOI:** 10.1371/journal.pone.0275682

**Published:** 2022-12-20

**Authors:** Kevin Montagne, Katsuko S. Furukawa, Yuki Taninaka, Brandon Ngao, Takashi Ushida

**Affiliations:** 1 Department of Mechanical Engineering, University of Tokyo, Bunkyo-ku, Tokyo, Japan; 2 Department of Bioengineering, University of Tokyo, Bunkyo-ku, Tokyo, Japan; University of Stuttgart, GERMANY

## Abstract

Osteoarthritis (OA) is the most common joint disease in older adults and is characterized by a gradual degradation of articular cartilage due to decreased cartilage matrix gene expression and increased expression of genes involved in protein degradation, apoptosis and inflammation. Due to the high water content of cartilage, one of the main physical stimuli sensed by chondrocytes is hydrostatic pressure. We previously showed that high pressure above 20 MPa induced gene expression changes in chondrocyte precursor cells similar to what is observed in OA. Micro-RNAs are small non-coding RNAs essential to many physiological and pathological process including OA. As the micro-RNA miR-155 has been found increased in OA chondrocytes, we investigated the effects of high pressure on the expression of the miR-155 host gene *Mir155hg*. The chondrocyte progenitor cell line ATDC5 was pressurized under hydrostatic pressure up to 25 MPa and the expression of *Mir155hg* or the resulting micro-RNAs were measured; pharmacological inhibitors were used to identify the signaling pathways involved in the regulation of *Mir155hg*. We found that *Mir155hg* is strongly and rapidly up-regulated by high, but not moderate, pressure in chondrocyte progenitor cells. This up-regulation likely involves the membrane channel pannexin-1 and several intracellular signaling molecules including PKC and Src. MiR-155-5p and -3p were also up-regulated by pressure though somewhat later than *Mir155hg*, and a set of known miR-155-5p target genes, including *Ikbke*, *Smarca4* and *Ywhae*, was affected by pressure, suggesting that *Mir155hg* may have important roles in cartilage physiology.

## Introduction

Osteoarthritis (OA) is the most common joint disease in older adults and is characterized by a gradual degradation of articular cartilage, formation of osteophytes, subchondral bone thickening, and synovial inflammation, leading to pain, fatigue and reduced mobility [1]. Although great progress has been done in the past two decades, due to the inability of cartilage to self-repair, treatment is often limited to stopping the progression of the disease and manage symptoms [2]. Though the exact cause isn’t known, several risk factors have been identified such as age, intensive physical activity, obesity, joint trauma and genetic predisposition [3]. So far, no cure has been found, but physical loading of the joints through moderate physical exercise has been shown to slow the progression of the disease [4,5], suggesting that mechanical stimulation is essential to cartilage maintenance and health. In vivo, joint loading is believed to be sensed by chondrocytes partly in the form of hydrostatic pressure (HP) typically ranging from 3–10 MPa [6], and application of low or moderate HP in vitro has been used to promote cartilage differentiation in tissue engineered cartilage [7,8], counteract the effects of OA or interleukin-1β on human chondrocyte morphology [9], and stimulate chondrocyte differentiation [10,11].

At the cellular level, OA is characterized by a decreased expression of cartilage markers such as SOX9, a transcription factor essential to chondrogenesis, and aggrecan, an essential component of the cartilage matrix [12], an increase in cartilage-degrading enzymes and inflammatory markers, and cell death [13]. High HP (24 MPa) has been shown to decrease proteoglycan production in human OA chondrocytes [11]. We previously showed in a mouse chondrocyte progenitor cell line that high HP (above 20 MPa) induced changes in gene expression similar to what is observed in OA cartilage, with a decrease in chondrocyte marker expression and an increase in stress-, inflammation- and apoptosis-related gene expression [14]. Among the modulated genes, many long non-coding RNAs were present.

Micro-RNAs are a class of small non-coding RNAs that play a critical role in gene regulation by binding to complementary sequences in target mRNAs leading to their translational repression or cleavage. Most micro-RNAs are processed from longer primary transcripts after formation of hairpin structures that are cleaved by the Drosha complex and exported from the nucleus; this hairpin is cleaved again by the Dicer complex, leaving only the stem of the hairpin, which is composed of a micro-RNA duplex, one strand of which forms a complex with several proteins such as Argonaute and binds to target mRNAs [15].

Many micro-RNAs are modulated by mechanical stimuli, including hydrostatic pressure [16]. In OA chondrocytes, moderate cyclical HP up-regulates several micro-RNAs essential for proper cartilage physiology leading to the down-regulation of several genes involved in cartilage degradation [17]. Among the mechano-sensitive micro-RNAs, miR-155 has been found to be down-regulated by low (1 to 5 MPa), cyclical HP [18] and up-regulated by high (24 MPa), continuous HP [19] in OA chondrocytes.

MiR-155-5p is a well characterized micro-RNA that plays essential roles in immunity, hematopoietic differentiation, cancer and inflammation [20]. In mice, it is processed from the mir-155 host gene *Mir155hg* (the third exon in the human *MIR155HG*), a long non-coding RNA, to produce two strands, the most abundant guide strand miR-155-5p and less abundant passenger strand miR-155-3p. Furthermore, Mir-155 is mechano-sensitive, as it is up-regulated by shear stress in endothelial cells [21]. However, the mechanisms involved in the *Mir155hg* regulation are still not well characterized.

As miR-155 expression has also been found to be slightly increased in OA chondrocytes [18], we applied high HP to chondrocyte progenitor cells to mimic a pro-arthritic environment and investigated the molecular mechanisms involved in regulating *Mir155hg* expression. A better understanding of the modulation of this gene may indeed shed some light on the factors contributing to the pathogenesis of OA. Therefore, after confirming that high pressure induced *Mir155hg* expression, inhibitor experiments were carried out that enabled us to identify some of the intracellular signaling molecules involved in this gene’s modulation.

## Materials and methods

### Reagents

Dulbecco’s Modified Eagle Medium (DMEM), trypsin and the antibiotic/antimycotic solution were purchased from Life Technologies Corporation, Grand Island, New York, USA. F-12 Nutrient Mixture (Ham) was from MP Biomedicals LLC, Solon, Ohio, USA. Fetal bovine serum (FBS) was from Nichirei Biosciences Inc., Tokyo, Japan. The TRPP3 and Na^+^/H^+^ exchanger inhibitor ethyl-isopropyl-amiloride (EIPA) was from Cayman Chemical and was added to the medium 30 min before pressurization at 50 μM. The MEK inhibitor U0126 was from Promega and was added to the medium 15 min before pressurization at 10 μM. The p38 inhibitor SB203580 was from Cayman Chemical and was added to the medium 15 min before pressurization at 10 μM. The JNK inhibitor SP600125 was from Wako and was added to the medium 15 min before pressurization at 10 μM. The broad heterotrimeric G protein inhibitor suramin was from Wako and was added to the medium 20 min before pressurization at 100 μM. The PKC (α, β, γ and ε) inhibitor bisindolylmaleimide XI was from Cayman Chemical and was added to the medium 30 min before pressurization at 2 or 10 μM. The broad spectrum PKC (α, β, γ, δ and ζ) inhibitor Gö6983 was from Cayman Chemical and was added to the medium 30 min before pressurization at 2 or 10 μM. The NF-κB inhibitor Ro106-9920 was from Abcam and was added to the medium 30 min before pressurization at 2, 5 or 10 μM. The antioxidant and NF-κB inhibitor caffeic acid phenethyl ester (CAPE) was from Cayman Chemical and was added to the medium 1 h before pressurization at 10 μM. The pannexin-1 hemichannel inhibitor Probenecid was from Wako and was added to the medium 1 h before pressurization at 3 mM. The ENaC channel and sodium-calcium exchanger inhibitor Benzamil was from Santa Cruz Biotechnology and was added to the medium 30 min before pressurization at 10 or 50 μM. The Src inhibitor PP2 was from Cayman Chemical and was added to the medium 10 min before pressurization at 10 μM.

### Cell culture

Mouse ATDC5 chondrocyte progenitor cells were obtained from the Japanese Collection of Research Bioresources Cell Bank (RCB0565) and cultured in DMEM/F12 (Life Technologies) supplemented with 5% of FBS in a humidified incubator under a 5% CO_2_ atmosphere.

### Cell pressurization

Hydrostatic pressure was applied for up to 48 h with a mechanical system described previously [14] and consisting of a cylindrical pressure chamber placed inside a 37°C water bath, and connected to a high-pressure cylinder actuated by a manual pump. The pressure cylinder can generate static pressures of up to 25 MPa in the chamber. Slightly above the physiological range of pressures, 25 MPa was chosen to induce changes similar to those observed in osteoarthritic chondrocytes [14]. Before pressurization, ATDC5 cells were seeded in 35 mm Petri dishes until they formed a monolayer, sealed in polyethylene bags (Seisan Nippon Sha, Ltd., Tokyo, Japan) filled with 15 mL of culture medium and placed in the pressure chamber where pressure was applied. The internal pressure of the chamber was monitored using a pressure sensor. Unpressurized cells were sealed in similar bags and inserted in a metal column of the same dimensions as the pressure chamber placed in the same water bath.

### Real-time PCR

Total RNA was extracted using Trizol (Life Technologies Corporation, Carlsbad, California, USA) according to the manufacturer’s instructions. For the measurements of *Mir155hg* or messenger RNAs, cDNA was synthesized from 500 ng of RNA using ReverTra Ace qPCR RT Master Mix with gDNA Remover (Toyobo Co., Ltd., Osaka, Japan). Real-time PCR was then carried out using Thunderbird SYBR qPCR Mix (Toyobo) in a StepOnePlus real-time PCR system (Applied Biosystems, Foster City, California, USA). *Rpl13a* was used as the reference gene. The primer sequences are listed in Table 1. For micro-RNA measurements, cDNA was first synthesized using the TaqMan MicroRNA Reverse Transcription Kit (Applied Biosystems) together with the specific primer for mmu-miR-155, mmu-miR-155* or U6 snRNA provided in TaqMan MicroRNA Assays (Applied Biosystems). After cDNA synthesis, the micro-RNAs and U6 snRNA, used as a reference, were quantified using the TaqMan Universal PCR Master Mix, No AmpErase UNG (Applied Biosystems) with the primers and MGB probe provided in the TaqMan MicroRNA Assays, all according to the manufacturer’s instructions.

**Table 1:** Primers used for real-time PCR.

Gene	Forward primer	Reverse primer	Amplicon size (bp)
*Rpl13a*	TCTGGAGGAGAAACGGAAGGA	GGTTCACACCAAGAGTCCATTG	151
*Fgf7*	CCATGAACAAGGAAGGGAAA	TTGACAGGAATCCCCTTTTG	166
*Foxo3*	GGTACCAGGCTGAAGGATCA	GAGAGCAGATTTGGCAAAGG	102
*Ikbke*	GGAAAATGGTCCCTTGGAGT	AAAGCCCCAGCACTTATCCT	130
*Mir155hg*	AAACCAGGAAGGGGAAGTGT	GTCAGTCAGAGGCCAAAACC	123
*Smarca4*	AACCAAAGCAACCATCGAAC	TCTCCAGGGCTGTGTCTCTT	104
*Socs1*	GAGCTGCTGGAGCACTACG	CACGGAGTACCGGGTTAAGA	160
*Wee1*	AGCGCAGAGCAGTTACGAAT	CTAGTGGCCATCCGATCTGT	140
*Ywhae*	GAGCGATACGACGAAATGGT	GCTCTTCTGGCTCCAATCAC	122

### Micro-RNA and lncRNA target prediction

Predicted targets of the mature miR-155-5p were searched using three prediction softwares: PicTar (https://pictar.mdc-berlin.de/cgi-bin/new_PicTar_mouse.cgi [22]), TargetScan (http://www.targetscan.org/mmu_72/ [23]), and miRDB (http://mirdb.org/ [24]), and one microRNA target database: DIANA-TarBase v8 (http://diana.imis.athena-innovation.gr/DianaTools/index.php?r=tarbase/index [25]). RNA species predicted to bind to *Mir155hg* were searched in the LncRRIsearch database [26].

### Rapid amplification of cDNA ends (RACE)

In order to identify the 5’ end (transcription start site) and 3’ end of *Mir155hg*, the FirstChoice RLM-RACE Kit was obtained from ThermoFisher Scientific Baltic UAB (Vilnius, Lithuania) and used according to the manufacturer’s instructions. RACE-PCR was carried out using pfu DNA polymerase (Promega, Madison, WI, USA). The 5’ RACE-PCR product was cloned in the pGL3-basic vector (Promega) between the XhoI and HindIII sites and the 3’ RACE-PCR product between the KpnI and XhoI sites, then sequenced using the RVprimer3 and GLprimer2 primers.

### Cell fractionation

In order to determine the localization of the *Mir155hg* RNA within the cell, the nuclei and cytosol were separated by cell fractionation: cells were scraped into phosphate-buffered saline, centrifuged at 700 g for 1 min, resuspended in 300 μL fractionation buffer (Hepes 20 mM pH 7.4, KCl 10 mM, MgCl_2_ 2 mM, EDTA 1 mM, EGTA 1 mM, DTT 1 mM) and incubated on ice for 15 min. The cells were then disrupted by passage 10 times through a 27-gauge needle. After incubation on ice for 20 min, the cells were centrifuged for 3 min and the supernatant (200 μL) was collected as the cytosolic fraction, from which RNA was isolated after addition of 1 mL of Trizol. The nuclei pellet was washed three times with fractionation buffer, then lyzed in Trizol.

### Statistics

All graphs show the mean +/- SEM of at least 3 independent experiments. For the inhibitor experiments, the effects of both pressure and inhibitor on gene expression were measured and, consequently, a two-way ANOVA followed by Tukey’s HSD test was used to detect whether the inhibitor significantly affected the pressure-induced gene modulation. For time-course and cell fractionation experiments, a one-way ANOVA was performed followed by Tukey’s HSD test. Statistical significance was assumed at p<0.05.

## Results

Little data is available on the mouse *Mir155hg* sequence. In order to clarify what lncRNA is present in ATDC5 cells, we first performed 5’ and 3’ RACE-PCR followed by sequencing of the PCR products. The results show that, in ATDC5 cells, the transcription start site is situated 55 bases upstream of the sequence found in the NCBI database and the 3’ end is about 839 bases downstream that reported in the NCBI database (S1 Fig). Furthermore, in ATDC5 cells, *Mir155hg* seems to have only one exon, and the intron predicted on the NCBI database isn’t spliced; indeed, PCRs on cDNA using various primers targeted along the *Mir155hg* sequence show that the amplicons are the size expected if the PCR were carried out on genomic DNA (S2 Fig).

**Fig 1:**
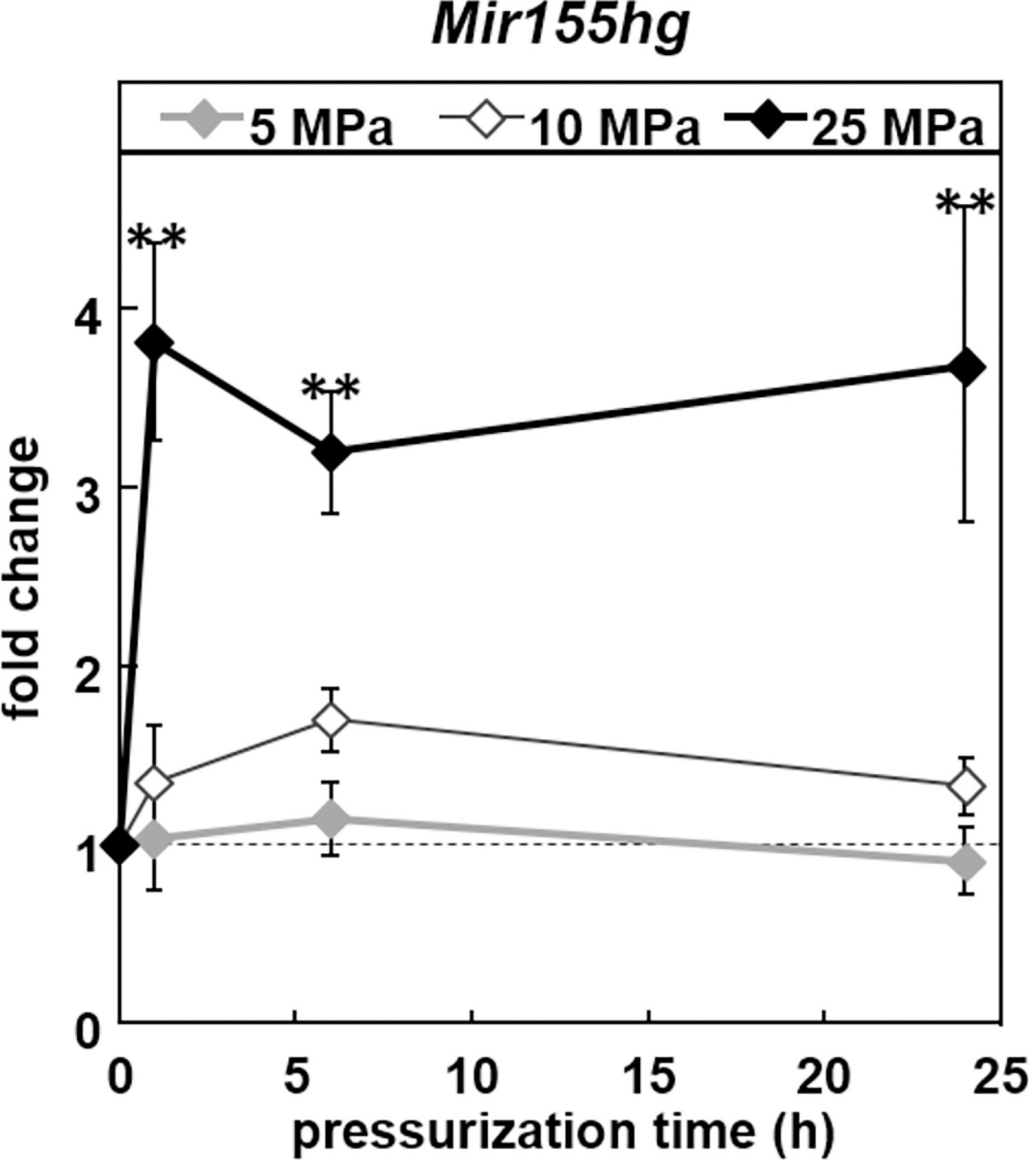
*Mir155hg* is up-regulated under high hydrostatic pressure. ATDC5 cells were pressurized for 0, 1, 6 or 24 h under 5, 10 or 25 MPa. *Mir155hg* expression was then measured by real-time PCR. The graph shows the mean +/- SEM of at least four independent experiments. The up-regulation of *Mir155hg* was significant under 25 MPa (one-way ANOVA followed by Tukey’s HSD, **:p<0.01 vs. no pressure).

In order to determine the effects of HP on *Mir155hg* expression, ATDC5 cells were pressurized for up to 24 h under 5 MPa (low pressure), 10 MPa (moderate pressure) or 25 MPa (high pressure). Real-time PCR showed that *Mir155hg* was significantly up-regulated only under high HP. The up-regulation was significant as early as 1 h after the start of pressurization and was maintained for at least 24 h (Fig 1).

**Fig 2:**
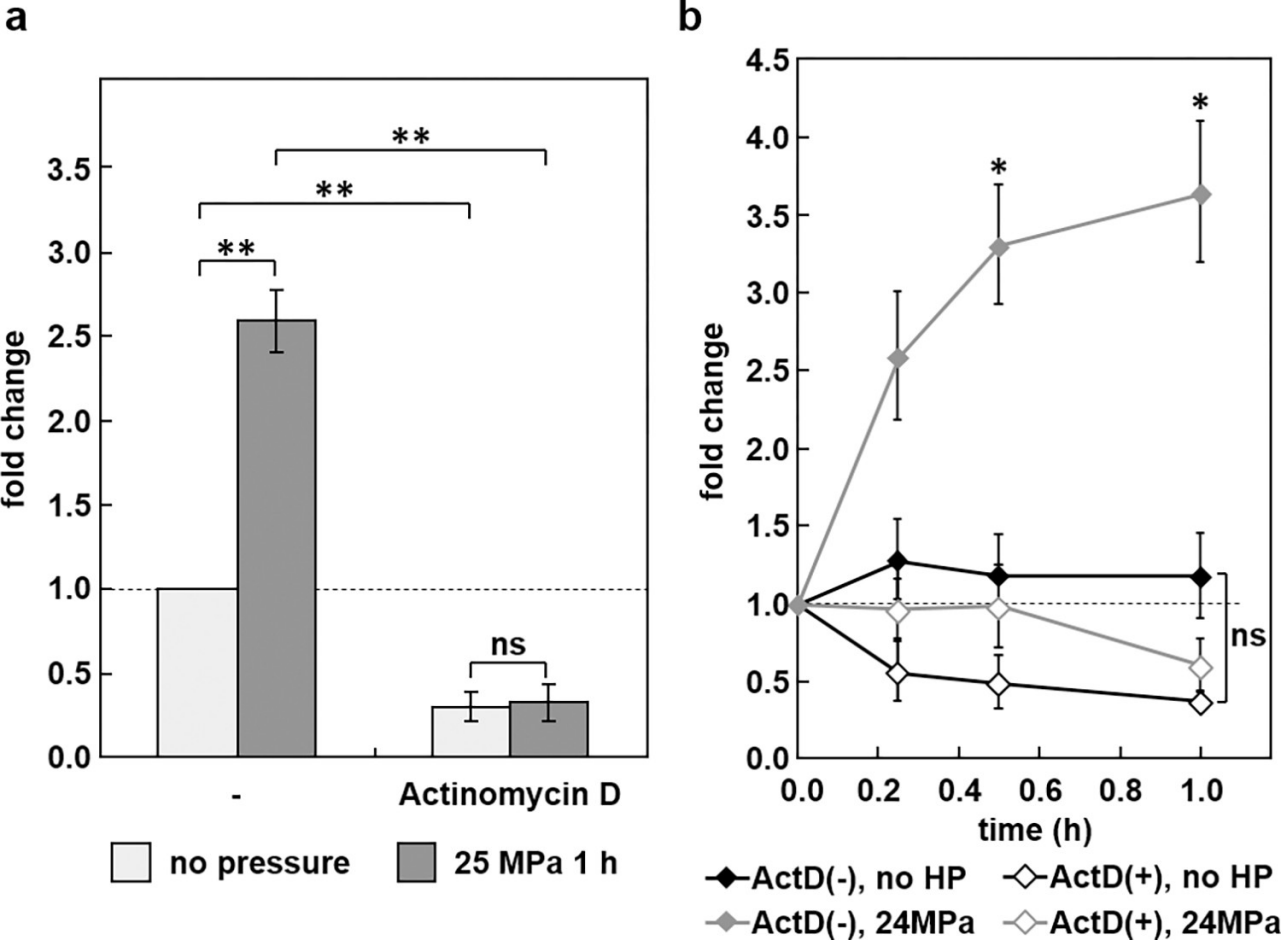
The up-regulation of *Mir155hg* is transcriptional. a) ATDC5 cells were pretreated with 4 μg/mL of actinomycin D for 15 min then pressurized, or not, for 1 h under 25 MPa. *Mir155hg* expression was then measured by real-time PCR. The graph shows the mean +/- SEM of four independent experiments. The up-regulation of *Mir155hg* under pressure was significantly inhibited by actinomycin D (two-way ANOVA followed by Tukey’s HSD, **:p<0.01). b) ATDC5 cells were pressurized for 0, 15, 30 or 60 min under 25 MPa in the presence (ActD(+)) or absence (ActD(-)) of 4 μg/mL of actinomycin D. *Mir155hg* expression was then measured by real-time PCR. The graph shows the mean +/- SEM of at least three independent experiments. Actinomycin D prevented the up-regulation of *Mir155hg* under pressure (two-way ANOVA followed by Tukey’s HSD, *:p<0.05 pressure without actinomycin D vs. other conditions).

To determine whether the *Mir155hg* up-regulation was due to increased transcription or reduced degradation of this long no-coding RNA, ATDC5 cells were pretreated with 4 μg/mL of the RNA transcription inhibitor actinomycin D for 15 min then pressurized, or not, for 1 h under 25 MPa. *Mir155hg* expression measurements by real-time PCR showed that the up-regulation of *Mir155hg* under pressure was significantly inhibited by actinomycin D, indicating that the *Mir155hg* up-regulation under HP is transcriptional (Fig 2A). Furthermore, actinomycin D treatment in the absence of pressure lead to a slow decrease in *Mir155hg* expression (normalized to *Rpl13a*) with a half-life of around 1 h. HP induced a significant up-regulation of *Mir155hg* after 30 min, which was inhibited by actinomycin D. Under pressure, the degradation of *Mir155hg* in actinomycin D-treated cells, relative to *Rpl13a*, was somewhat slower than in unpressurized cells but not significantly so (Fig 2B); the short pre-treatment time may not have been sufficient to entirely block the increase in *Mir155hg* transcription induced by HP.

**Fig 3:**
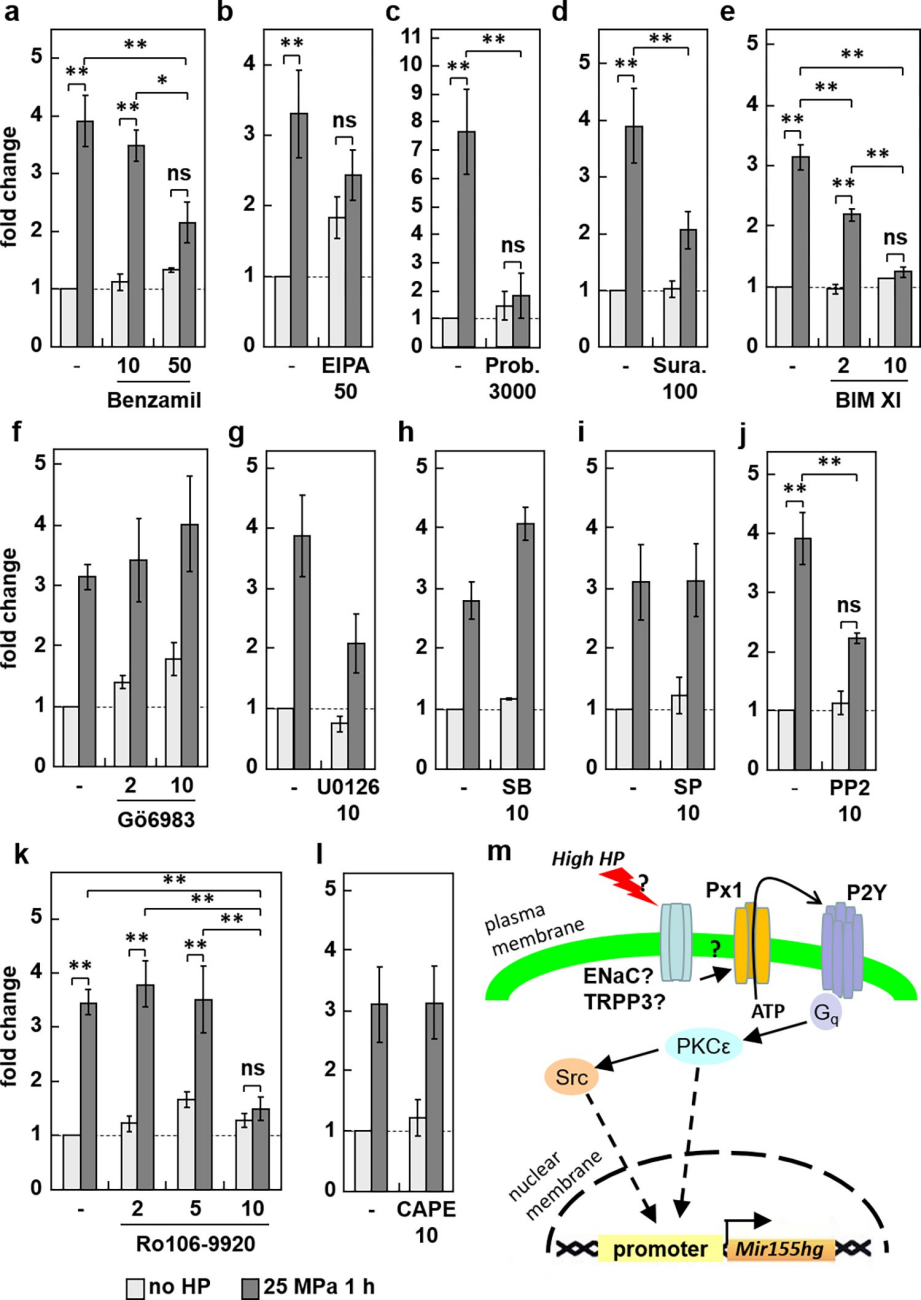
Effect of various signaling inhibitors on the *Mir155hg* up-regulation by HP. ATDC5 cells were pre-treated with a) the ENaC channel and sodium-calcium exchanger inhibitor benzamil, b) the TRPP3 channel inhibitor EIPA, c) the pannexin-1 inhibitor probenecid (Prob.), d) the broad G protein inhibitor suramin (Sura.), e-f) the PKC inhibitors bisindolylmaleimide XI (BIM XI) or Gö6983, g-i) the ERK1/2, p38 or JNK MAP kinase inhibitors U0126, SB203580 (SB) or SP600125 (SP), j) the Src inhibitor PP2, or k-l) the NF-κB inhibitors Ro106-9920 and CAPE at the indicated concentrations (in μM). After this pre-treatment, the cells were pressurized, or not, for 1 h under 25 MPa. *Mir155hg* expression was then measured by real-time PCR. The graphs show the mean +/- SEM of at least three independent experiments. Statistical significance was assessed by performing a two-way ANOVA followed, only when the ANOVA p-value was <0.05, by Tukey’s HSD, the asterisks indicate the statistical significance of the differences between the indicated conditions: *:p<0.05, **:p<0.01) schematic diagram of the possible mechanotransduction pathway leading from HP mechano-sensing to *Mir155hg* transcription.

In order to identify the intracellular signaling pathways involved in the *Mir155hg* up-regulation, we performed a series of inhibitor experiments targeting the TRPP3 channel and Na^+^/H^+^ exchanger, the ENaC channel and sodium-calcium exchanger (NCX), pannexin-1, heterotrimeric G proteins, the MAP kinases, various PKC isoforms, Src, and NF-κB. Before pressurization, ATDC5 cells were therefore pretreated with the respective inhibitors as detailed in the previous section. *Mir155hg* expression measurements showed that benzamil, EIPA, probenecid, suramin, BIM XI, and PP2 significantly inhibited the up-regulation of *Mir155hg* by pressure (Fig 3) suggesting that ENaC or the NCX exchanger, TRPP3 or the Na^+^/H^+^ exchanger, pannexin-1, heterotrimeric G proteins and Src are involved in the modulation of *Mir155hg*. The MAP kinases, however, do not seem to be. Regarding PKC isoforms, Gö6983, which does not inhibit PCKε [27], had no effect, whereas BIM XI, which inhibits PKCε among others, did inhibit the *Mir155hg* up-regulation, suggesting that PKCε is involved in this modulation. Concerning NF-κB, CAPE had no effect on the *Mir155hg* up-regulation and only the highest dose of Ro106-9920 did, suggesting that inhibited NF-κB may not be important for *Mir155hg* modulation under HP. The inhibitor experiments thus point to a possible transduction pathway leading from mechanical activation by hydrostatic pressure of a yet unidentified mechanosensor to ATP release and reception, activation of heterotrimeric G proteins, PKCε, and Src, and increased transcription of the *Mir155hg* gene (Fig 3M).

**Fig 4:**
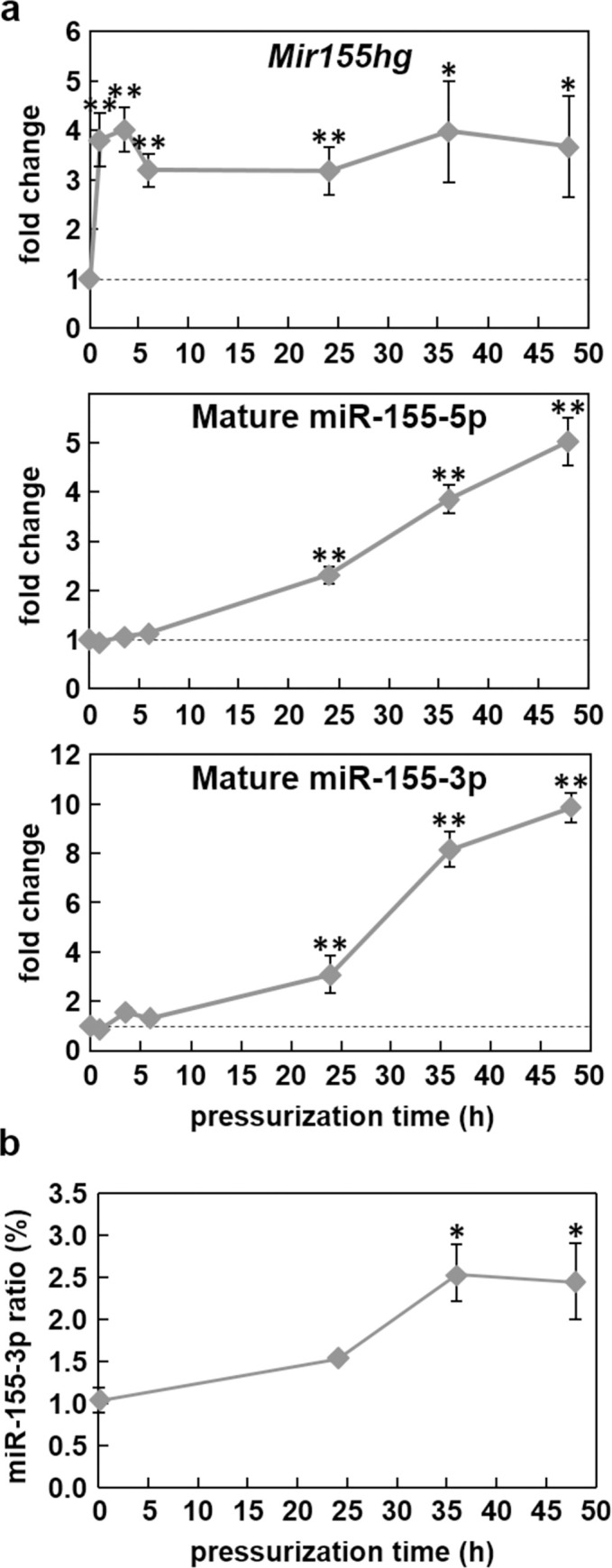
a) *Mir155hg* and the mature miR-155 are up-regulated under high hydrostatic pressure. ATDC5 cells were pressurized for 0, 1, 4, 6, 24, 36 or 48 h under 25 MPa. *Mir155hg*, miR-155-5p and miR-155-3p expression was then measured by real-time PCR. The graph shows the mean +/- SEM of at least four independent experiments (one-way ANOVA followed by Tukey’s HSD, *:p<0.05 and **:p<0.01 vs. no pressure). b) miR-155-3p/miR-155-5p ratio in pressurized ATDC5 cells. The graph shows the mean +/- SEM of three independent experiments (one-way ANOVA followed by Tukey’s HSD, *:p<0.05 pressurized vs. no pressure).

As *Mir155hg* was rapidly and strongly up-regulated by pressure, we investigated the expression pattern of the mature micro-RNAs produced from *Mir155hg*, miR-155-5p and miR-155-3p. ATDC5 cells were pressurized for up to 48 h under 25 MPa and the various expressions were measured by real-time PCR. *Mir155hg* was up-regulated as early as 1 h after pressurization but miR-155-5p and miR-155-3p were only significantly induced after at least 24 h (Fig 4A). Taking into account the PCR efficiencies of miR-155-5p and miR-155-3p, the relative abundance of the two micro-RNAs was calculated (Fig 4B). The 3p/5p ratio was estimated at around 1 to 3%; as the expression of miR-155-3p increased faster under pressure than that of miR-155-5p, the 3p/5p ratio increased significantly after 36 or 48 h under HP, but still remained low at around 3% of total miR-155.

**Fig 5:**
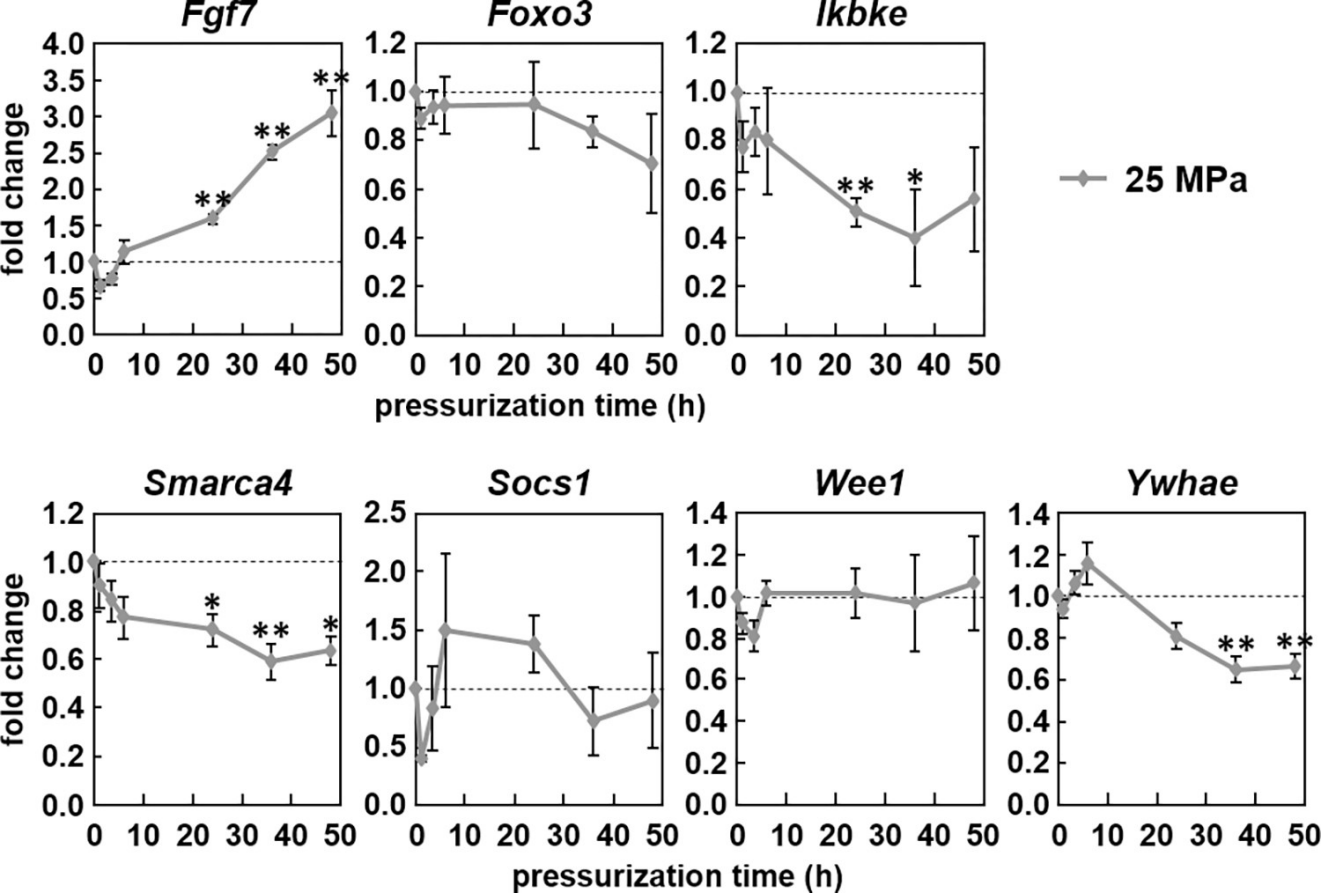
Expression of miR-155 targets under pressure. ATDC5 cells were pressurized for up to 48 h under 25 MPa. *Fgf7*, *Foxo3*, *Ikbke*, *Smarca4*, *Socs1*, *Wee1* and *Ywhae* expression was then measured by real-time PCR. The graph shows the mean +/- SEM of at least three independent experiments. (one-way ANOVA followed by Tukey’s HSD, *:p<0.05, **:p<0.01 pressurized vs. no pressure).

As the mature micro-RNAs were also up-regulated, though much later than *Mir155hg*, we measured the expression over time of several known or predicted miR-155-5p target genes in ATDC5 cells submitted to 25 MPa for up to 48 h (Fig 5). A search using three micro-RNA target prediction softwares and one target database yielded six mRNAs that were predicted as miR-155-5p targets in at least three of them: *Fgf7*, *Ikbke*, *Smarca4*, *Socs1*, *Wee1* and *Ywhae*. A literature search further found that *Foxo3* was a target in two previous reports (Table 2). Consequently, ATDC5 cells were pressurized for up to 48 h under 25 MPa and the expression of those seven genes was measured by real-time PCR. *Ikbke*, *Smarca4* and *Ywhae* were down-regulated after at least 24 h of pressurization. *Foxo3*, *Socs1* and *Wee1*, however, were unchanged by pressure and *Fgf7* was even up-regulated after 24 h.

**Table 2:** Messenger RNAs targeted by miR-155 and the target sequences in the 3’UTR. D, M, P and T indicate the target RNAs found respectively with the DIANA-TarBase v8 database, the miRDB, PicTar, and TargetScan softwares. Other references are cited in the reference column.

miR-155-5p sequence (seed underlined)	5’-UUAAUGCUAAUUGUGAUAGGGGU-3’		
Targeted gene	Targeted sequence (underlined)	Position in the 3’UTR	Reference
*Fgf7*	…UUAAAAGACUGCAUUAAAGAAAGAUUU…	185 to 191	D, M, P, T, [28]
	…CUGUGAUUACAGCAUUAAACUCUACUUU…	880 to 887	
*Foxo3*	…AACUGUGACUGCAUUAGAGGUGUGUU…	3051 to 3056	[29,30]
*Ikbke*	…CCAACAAACUAGCAUUACUUUGACUGU…	146 to 152	D, M, P, T, [31]
*Smarca4*	…AGUCUCUACCAGCAUUAACUGUCUAGAG…	188 to 195	M, P, T, [32]
*Socs1*	…GCUGUGCCGCAGCAUUAAGUGGGGGCG…	20 to 27	D, M, P, T, [33,34]
*Wee1*	…CUGUAAAACUAGCAUUAAACAAUCAUUG…	590 to 597	D, M, P, T, [35,36]
*Ywhae*	…AGGCUUUUUCAGCAUUACUGUAUUGUC…	307 to 313	D, P, T

**Fig 6:**
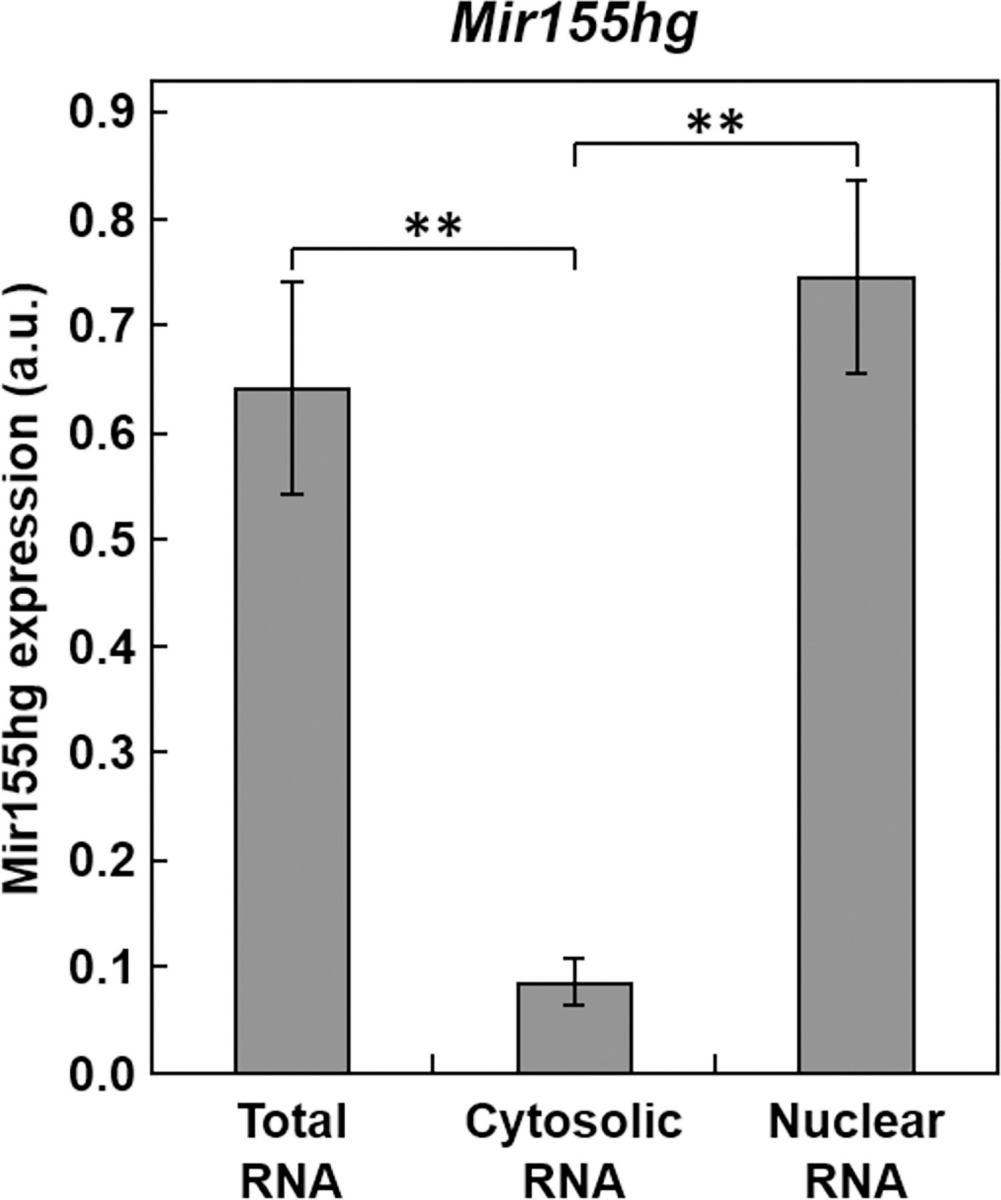
The localization of *Mir155hg* is predominantly nuclear. Total, cytosolic and nuclear RNA was extracted from ATDC5 cells and *Mir155hg* expression was then measured by real-time PCR and normalized to *Rpl13a* expression. The graph shows the mean +/- SEM of five independent experiments (one-way ANOVA followed by Tukey’s HSD, **: p<0.01).

As Mir155hg is known to sometimes function independently of miR-155 production [37], and since many long non-coding RNAs are known to be located in the nucleus where they directly modulate gene expression, we determined the localization of *Mir155hg* by cell fractionation followed by real-time PCR. The results showed that *Mir155hg* is predominantly located in the nucleus (Fig 6), suggesting that, besides its potential effects on miR-155 target gene expression, *Mir155hg* could also function in the nucleus independently of micro-RNA production.

## Discussion

We have shown that high HP induces a strong and rapid increase in *Mir155hg* expression, followed by an increase in miR-155 expression after 24 h. Cheleschi et al. also found an increase in miR-155 expression in OA chondrocytes exposed to 24 MPa for 3 h [19]. The difference could be due to the more mature phenotype of the chondrocytes, or to changes induced by OA in chondrocytes affecting mechano-sensitive molecules or structures: for instance, OA increases the number and length of primary cilia, a mechano-sensitive structure, in OA cartilage [38]. No significant effect was observed under 5 MPa. De Palma et al. [18], however, showed that miR-155 is down-regulated by low HP (1 to 5 MPa) in chondrocytes from OA patients. The difference may be due to the cyclical nature of De Palma et al.’s pressurization protocol, as low, cyclical HP is known to have overall beneficial effects on chondrocytes and tissue engineered cartilage [7]. Although ATDC5 are a trusted and convenient model of chondrocyte progenitor cells, mature chondrocytes may better reflect the changes observed *in vivo* under mechanical stress. Data from patient samples, such as in situ hybridization images, may also help determine whether *Mir155hg* is dysregulated in OA cartilage, and will have to be obtained in future studies.

As the *Mir155hg* up-regulation was found to involve an increase in transcription, we sought to identify the intracellular molecules involved in this gene’s regulation. Inhibitor experiments were carried out and point to a mechanotransduction mechanism, by which high HP triggers signaling at the membrane; candidate mechanotransducers are the ENaC or TRPP3 channels, as benzamil and EIPA significantly inhibited the *Mir155hg* up-regulation. Both of these channels are known to be mechanosensitive [39,40].

Inhibition of the pannexin-1 hemichannel, which allows the passage of small molecules such as ATP, inhibited the *Mir155hg* up-regulation under HP, suggesting that ATP release, and probably autocrine or paracrine downstream signaling, participates in the mechanotransduction pathway. How ENaC activation leads to ATP release is unclear but blocking ENaC with amiloride has been shown to inhibit ATP release from urothelial cells under pressure [41] or from keratinocytes under low pH [42].

The likely next step after ATP release probably involves ATP reception by P2Y-type receptors and activation of heterotrimeric G proteins, as suramin significantly inhibited the effect of HP on *Mir155hg* expression. The main P2Y receptors that bind ATP (P2RY2 and P2RY11) typically activate the G_q_ alpha subunit leading to activation of phospholipase C then PKC [43]. The involvement of PKC in the regulation of *Mir155hg* expression has previously been reported in lymphoma cells [44], though the PKC isoform involved was not identified. PKCε, a novel PKC isoform, is activated by mechanical stimulation [45] or oxidative stress [46]. We previously showed that high HP up-regulates genes involved in cellular stress [14], though the PKC isoforms involved are still under investigation. Src inhibition suggested that Src was involved in the *Mir155hg* up-regulation under pressure, a finding that is supported by the fact that PKCε can activate Src in cancer cells [47]. The fact that Src inhibition did not completely prevent *Mir155hg* induction by HP suggests that PKCε may activate other effectors.

Surprisingly, NF-κB does not seem to be involved in the up-regulation of *Mir155hg* expression under HP, despite several studies showing that NF-κB modulates miR-155 expression by binding to the *Mir155hg* promoter [48,49]. Therefore, several points remain to be addressed: what other transcription factors participate in the pressure-induced *Mir155hg* up-regulation, and what promoter elements are activated during this process.

The meaning of the *Mir155hg* up-regulation under pressure is still under investigation. Approximately 24 h after pressurization, possibly due to a low processing microprocessor activity in ATDC5 cells, the expression of both micro-RNAs resulting from *Mir155hg* processing were finally significantly up-regulated. The canonical micro-RNA miR-155-5p has been shown to modulate many different genes, including the seven tested in this study. *Fgf7* was found to be up-regulated under pressure and has been found to be up-regulated in OA meniscal cells [50]. Furthermore, *Smarca4* expression, which was significantly down-regulated under HP, is reduced in a mouse model of OA [51]. Those two genes therefore behave under high HP as in cells from OA cartilage. *Ywhae*, however, was down-regulated under HP. Bone cells submitted to compression or extracted from OA patients have been shown to release the resulting protein, which, in chondrocytes, induces the expression of catabolic enzymes like MMP-3 or MMP-13 [52]. The down-regulation of *Ywhae* under high HP may be a protective mechanism against excessive cartilage degradation. Similarly, Long et al. found that an increase in *Mir155hg* expression led to a simultaneous decrease in *Ikbke* and *Mmp3* expression, though they did not show a direct link between those two genes [31], suggesting another protective mechanism by *Mir155hg* against excessive cartilage degradation.

The other known miR-155-5p target genes *Socs1*, *Wee1* and *Foxo3*, however, remained unaffected by high HP. If *Socs1* expression is known to be unaffected by osteoarthritis [53], *Foxo3* expression is down-regulated in aging and osteoarthritic cartilage [54]; furthermore, a study measured a low *Wee1* expression in OA patients, though that study did not show expression levels in healthy individuals [55]. This may simply reflect the fact that gene expression modulation can be quite complex and rely on more than a single micro-RNA: for instance, FOXO3, a product of the *Foxo3* gene, which is a target of miR-155-5p, is targeted for degradation by IKBKE, the product of the *Ikbke* gene down-regulated by miR-155-5p [56].

We did not focus in this study on miR-155-3p target genes, but we measured that the calculated 3p to 5p ratio was around 1 to 3% in ATDC5 cells under pressure. As the 3p strand of the miR-155 micro-RNA has recently been shown to have multiple roles in various contexts, including cancer, inflammation and bone growth [57], investigating the effects of pressure on miR-155-3p target genes may be useful to uncover new functions of *Mir155hg* in cartilage biology.

As *Mir155hg* up-regulation does not always lead to miR-155 up-regulation in other cell types [44], it is possible that *Mir155hg* may have other functions non-related to the production of miR-155. Knowing the localization of a lncRNA gives an indication on its potential function; lncRNAs retained in the nucleus are known to modulate gene expression by sequestering RNA-binding proteins or micro-RNAs, or recruiting transcription factors and chromatin modifiers to gene promoters [58]. *Mir155hg* has also been found to function independently of miR-155 for instance in infection [37].

Regarding potential interactions with other RNA species, a search on the LncRRIsearch database showed that *Mir155hg* was predicted to bind to at least 4 RNA species in both human and mouse: the mRNA *Fam98b* and the lncRNAs *H19*, *Kcnq1ot1* and *Neat1*. *H19* has been found to be up-regulated in cartilage tissue from OA patients [59] and, in human chondrocytes, is controlled by SOX9 and controls the expression of the chondrocyte marker *Col2a1* [60]. *Kcnq1ot1* is an antisense lncRNA that regulates imprinted gene expression by binding to chromatin and changing its structure [61]. Like *H19*, it is involved in Beckwith-Wiedemann syndrome, but its function in cartilage is still unknown.

Regarding protein interactions, a database search (http://starbase.sysu.edu.cn/) showed that *Mir155hg* is expected to bind both in humans and mice to FUS, as well as PTBP1 and MSI1. The fact that *Mir155hg* is also predicted to bind to FUS is interesting: in cardiomyocytes, *Kcnq1ot1* has also been found to bind to FUS, thus increasing its expression and promoting apoptosis [62]; furthermore, *Neat1* and FUS are essential components of nuclear paraspeckles, nuclear bodies likely involved in regulating gene expression [63]. There may exist a complex interplay between FUS, *Mir155hg* and those other two lncRNAs. Identifying other RNA species that bind to *Mir155hg* and investigating whether *Mir155hg* actually binds to FUS directly or in a complex with other lncRNAs may be a promising step to uncover new *Mir155hg* functions.

## Conclusion

In summary, the results show that *Mir155hg* is strongly up-regulated by high, but not moderate, HP in ATDC5 cells. This up-regulation seems to involve membrane channels such as pannexin-1 and several intracellular signaling molecules including PKC and Src. While *Mir155hg* is strongly up-regulated in as little as 1 h after pressurization, the known micro-RNAs originating from *Mir155hg* are up-regulated after at least 24 h, and three known miR-155-5p target genes (*Ikbke*, *Smarca4* and *Ywhae*) were down-regulated at that time. These data indicate that the *Mir155hg* up-regulation could be an important factor in the inflammatory process that takes place during OA, and further studies on the modulation or cellular localization of this lncRNA in cartilage samples are warranted, as *Mir155hg* could serve as a new marker of the disease. Other potential functions of *Mir155hg* independent of micro-RNA production cannot be ruled out and may suggest new mechanisms for the onset or the progression of OA.

## Supporting information

S1 Fig *Mir155hg* RACE and sequencing of 5’ and 3’ ends. The sequence marked in yellow is the sequence of the 5’ RACE-PCR product; the sequence marked in grey is the sequence of the 3’ RACE-PCR product. The exons 1 and 2 reported in the NCBI database (NCBI Reference Sequence NR_132106.1) are underlined. The pre-miR-155 sequence is in bold. The forward (Fx) and reverse (Rx) PCR primers used for S2 Fig are respectively colored in red and blue.
(TIF)

S2 Fig Gel electrophoresis of PCR products generated along the *Mir155hg* cDNA sequence. PCRs were carried out using the Taq DNA Polymerase with Standard Taq Buffer (New England Biolabs) on ATDC5 cDNA using the primers shown in S1 Fig. The primers were designed to produce overlapping products covering the whole *Mir155hg* sequence (between primers F1 and R5). Two ladders were used; the lengths of some of the bands, in base pairs (bp), are shown on the side of each image. Under each lane, the primers used and the size of each amplicon expected if the PCR were carried out on genomic DNA is shown.
(TIF)
