# Supplementary figures and images for: Modulation of the long non-coding RNA Mir155hg by high, but not moderate, hydrostatic pressure in cartilage precursor cells
Source: PLoS One. 2022 Dec 20;17(12):e0275682. doi: 10.1371/journal.pone.0275682 (PMC9767356; doi:10.1371/journal.pone.0275682)

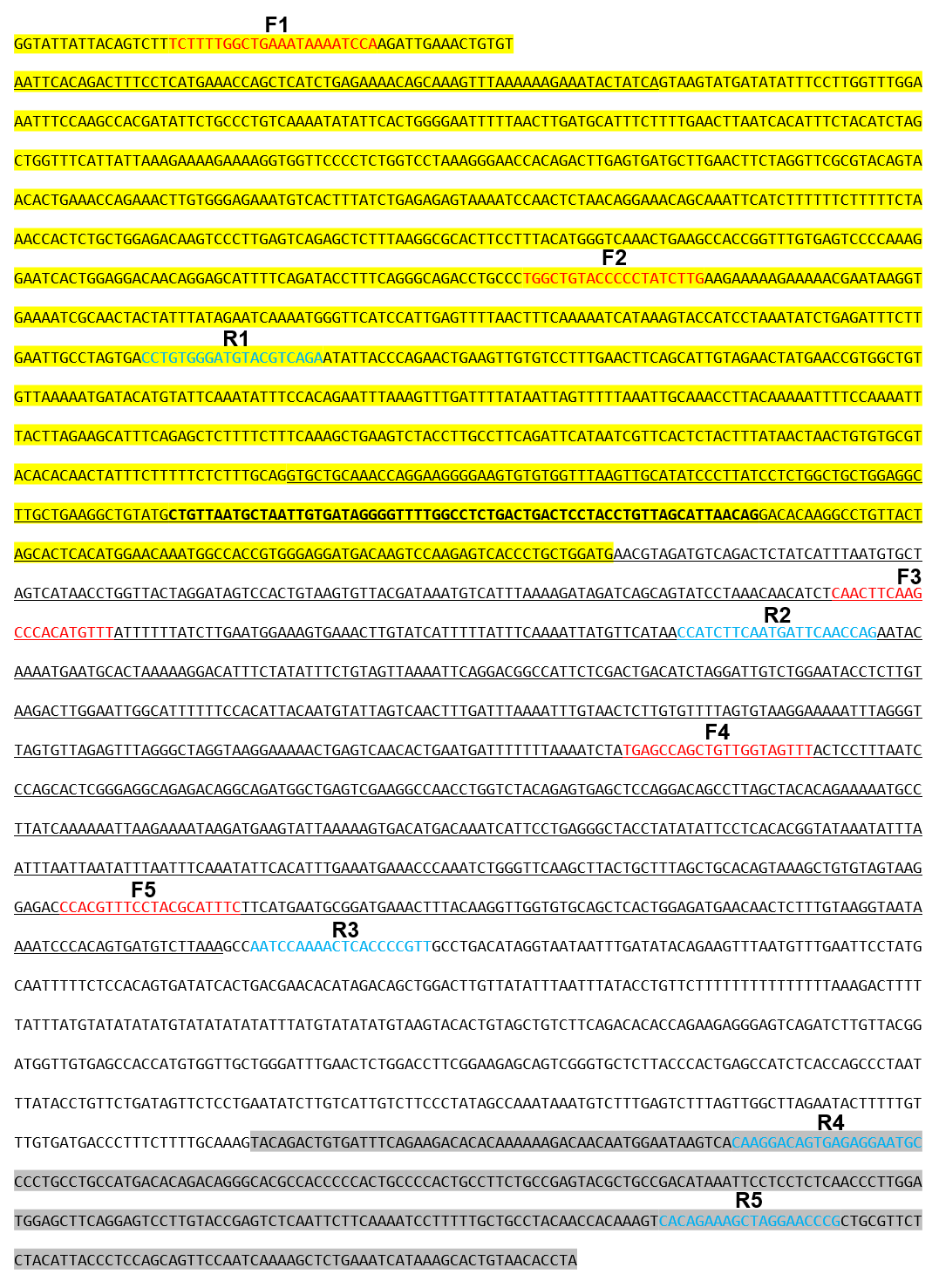

Supplement: S1 Fig — The sequence marked in yellow is the sequence of the 5’ RACE-PCR product; the sequence marked in grey is the sequence of the 3’ RACE-PCR product. The exons 1 and 2 reported in the NCBI database (NCBI Reference Sequence NR_132106.1) are underlined. The pre-miR-155 sequence is in bold. The forward (Fx) and reverse (Rx) PCR primers used for S2 Fig are respectively colored in red and blue. (TIF) [file pone.0275682.s001.tif]

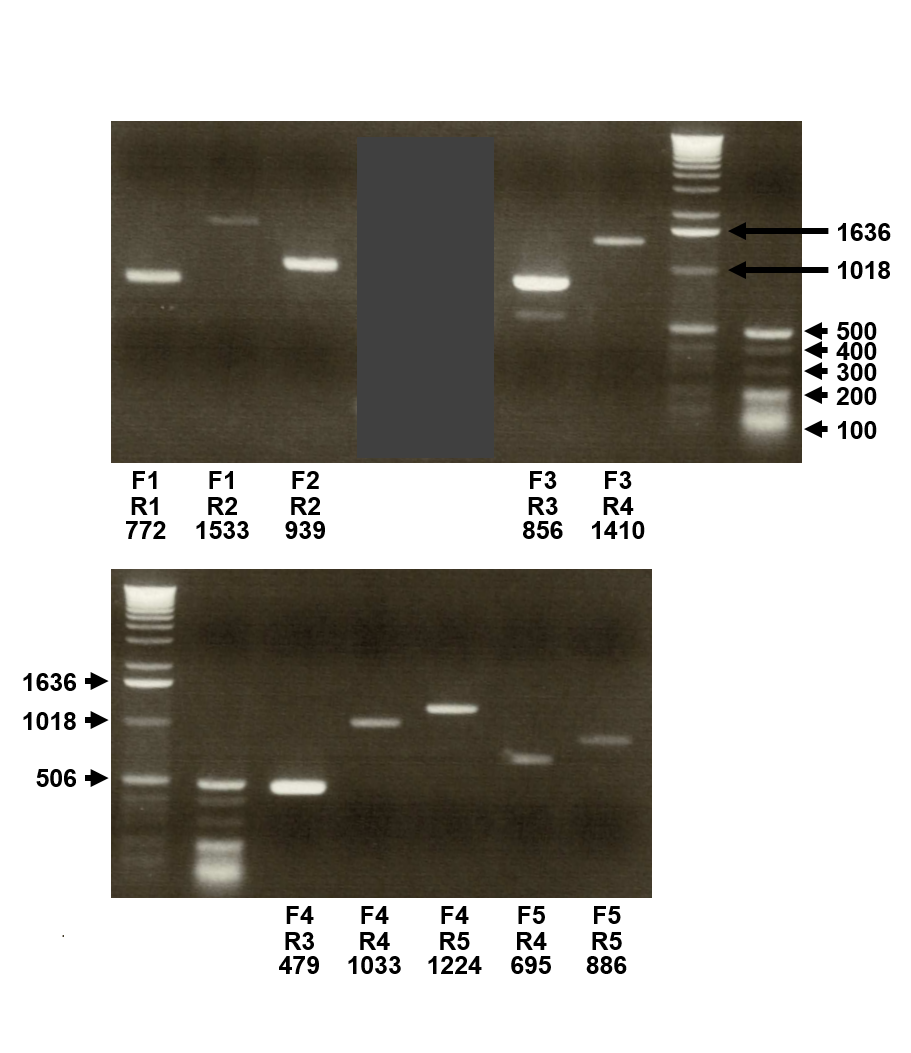

Supplement: S2 Fig — PCRs were carried out using the Taq DNA Polymerase with Standard Taq Buffer (New England Biolabs) on ATDC5 cDNA using the primers shown in S1 Fig. The primers were designed to produce overlapping products covering the whole Mir155hg sequence (between primers F1 and R5). Two ladders were used; the lengths of some of the bands, in base pairs (bp), are shown on the side of each image. Under each lane, the primers used and the size of each amplicon expected if the PCR were carried out on genomic DNA is shown. (TIF) [file pone.0275682.s002.tif]
